# A Maximum Feasible Subsystem for Globally Optimal 3D Point Cloud Registration

**DOI:** 10.3390/s18020544

**Published:** 2018-02-10

**Authors:** Chanki Yu, Da Young Ju

**Affiliations:** 1 Department of Media Technology, Sogang University, Seoul 04107, Korea; ckyu@sogang.ac.kr; 2 Yonsei Institute of Convergence Technology, Yonsei University, Incheon 21993, Korea

**Keywords:** pairwise three-dimensional (3D) registration, 3D measurement, point cloud alignment, outlier removal, maximum feasible subsystem

## Abstract

In this paper, a globally optimal algorithm based on a maximum feasible subsystem framework is proposed for robust pairwise registration of point cloud data. Registration is formulated as a branch-and-bound problem with mixed-integer linear programming. Among the putative matches of three-dimensional (3D) features between two sets of range data, the proposed algorithm finds the maximum number of geometrically correct correspondences in the presence of incorrect matches, and it estimates the transformation parameters in a globally optimal manner. The optimization requires no initialization of transformation parameters. Experimental results demonstrated that the presented algorithm was more accurate and reliable than state-of-the-art registration methods and showed robustness against severe outliers/mismatches. This global optimization technique was highly effective, even when the geometric overlap between the datasets was very small.

## 1. Introduction

The registration problem of partially overlapped three-dimensional (3D) surfaces is a fundamental issue in computer vision, computer graphics, and robotics. Despite significant research efforts in recent decades, solving the 3D Euclidean registration between two point clouds remain challenging when the rough transformation between point cloud sets is unknown or there is severe noise and outliers in the point cloud sets. This issue is closely related to several areas, including 3D scene reconstruction [1], object instance recognition [2], 3D pose estimation [3], simultaneous location and mapping (SLAM) [4], digital cultural heritage [5], and robot grasping systems [6]. 

The focus of this paper is pairwise registration. The iterative closest point (ICP) algorithm is widely employed for registering pairwise dense 3D data sets in an alternating, iterative manner. A transformation is estimated that minimizes the Euclidean distances between the pairs of closest points, in the least squares sense, by alternating between (1) building closest-point correspondences under the current transformation, and (2) estimating the transformation with current correspondences until convergence. The *L*_2_ error metric defined in ICP corresponds to the maximum likelihood estimate of transformation when the matching noise between two point clouds is Gaussian [7]. Chanpleboux et al. [8] discussed the problems caused by the small overlap between two point clouds and the initial estimate parameters being too far from the true solution. Given the data scanned from one part of an object and the corresponding data from a different side of the same object, the distance minimization produces the wrong estimate because non-overlapped points are treated as outliers in the ICP process. The main limitation of ICP is that it requires a preprocessing procedure of rough alignment between two point clouds because it is sensitive to the initial conditions and easily trapped in local minima, owing to the optimization’s non-convexity. Nevertheless, ICP and its variants are very popular and have been applied in numerous real-world settings due to their simplicity and ease of use. In addition, several variants of the original ICP have been proposed to improve robustness and deal with outliers [9,10,11]. Trimmed ICP, which employs the least trimmed squares for an error metric instead of least squares defined in the ICP, is robust to outliers and mismeasurements. When the overlap of two point clouds is more than 50%, this method is applicable because the least trimmed squares metric has a breakdown point of 50%. Levenberg–Marquardt (LM) ICP was introduced to improve robustness to the poor initialization of estimate parameters, optimizing the error metric with the LM algorithm instead of the linear least square method [11]. 

The pairwise registration approach is roughly classified into two groups: dense registration approaches, such as ICP, and feature-based registration approaches. Various 3D descriptors were introduced to establish correspondences between point cloud sets for 3D feature-based registration and object instance recognition, including spin images, point feature histograms, and signature of histograms of orientations (SHOT) descriptors [12,13,14]. Feature-based approaches are usually followed by ICP for dense refinement. Horn et al. [15] presented a closed-form solution for 3D pairwise registration with known point-to-point correspondences between two point clouds. When the correspondences are not firmly established, on the other hand, this technique is commonly used in conjunction with the random sample consensus (RANSAC) algorithm [16,17]. RANSAC is one of the most renowned and robust algorithms for estimating model parameters from data contaminated with noise and outliers. Although this method works well in practice, it sometimes fails to correctly estimate parameters and does not guarantee a globally optimal solution. Therefore, registration results may change on each trial, given the algorithm’s randomized nature. In other words, some outliers may remain [18].

Outlier removal algorithms that overcome the unpredictability of RANSAC using a global optimization approach have been presented, such as the maximum feasible subsystem (MaxFS) and consensus set maximization (CSM) algorithms [19,20]. The latter algorithm, presented by Li, is a robust algorithm for outlier removal in geometric vision problems [20]. Meanwhile, Yu et al. showed that the problem of outlier removal in photometric stereo can be viewed in terms of MaxFS [20,21]. These approaches are applied to several linear problems, including geometric and photometric vision problems, and are always guaranteed to find the maximum number of inliers [20,21,22,23].

Furthermore, research has been conducted on 3D registration based on a global optimization approach and most of the global optimization approaches are based on a branch-and-bound strategy [24,25,26]. The branch-and-bound strategy is a deterministic algorithm for global optimization in nonconvex problems, including combinatorial problems, which should guarantee provision of a global optimal solution in a reliable manner. It is a divide-and-conquer approach to find a global solution, and the key idea of this approach is to divide the original problem into smaller sub-problems by branching the parameter spaces then optimizing individually for each sub-problem, which should be easier to solve than the original problem [27,28]. In addition, the branch-and-bound algorithm requires an upper bounding function and a lower bounding function for the given problem. It may be noted that most branch-and-bound algorithms focus the investigation of the development of the lower bounding function on the given problem and employ a well-known local optimization with a low computational cost to compute the upper bounding function. Li and Hartley [26] present a global optimization algorithm for solving a point cloud registration problem with unknown point correspondences, which yields a provably global solution under the assumptions that two point clouds have the same size and the transformation is a pure rotation with no translation. This method uses a Lipschitzized, *L*_2_, error metric function minimized by a branch-and-bound method to search the rotation parameter space of the special orthogonal group, *SO*(3). Globally optimal ICP (Go-ICP) was introduced to address the issue of local minima in ICP. Go-ICP is based on a branch-and-bound scheme and provides a globally optimal solution to 3D Euclidean registration of two point clouds under the point-to-point *L*_2_ error metric defined in ICP and the trimmed *L*_2_ error metric, similar to the trimmed ICP to tackle outliers [24]. In other words, this work always yields the same registration solution, regardless of any initial guesses. In [24], the branching and searching techniques of the rotation parameter space of *SO*(3) proposed in [29] are employed and any rotations are represented by exploiting the angle-axis representation, which is also extended to search the parameter space of the special Euclidean group, *SE*(3), and derive the lower and upper bounds of the 3D registration error. Nevertheless, this method shows a poor fitting quality when the overlapping surface area between point clouds is small. A branch-and-bound method has been developed to solve the 3D Euclidean registration problem in the presence of incorrect correspondences [25]; however, it assumes that the translation is known and the upper and lower bounds of the angular registration error are calculated by the method presented in [29]. While angle-axis representation has been exploited in previous work [24,25] related to branch-and-bound approaches, in this paper, the quaternion is exploited to represent any rotation.

Branch-and-bound approaches guarantee the global optimality of the solution; however, the branch-and-bound algorithm can sometimes be very slow since it requires an intensive computational cost that grows exponentially with the data and problem size. Briales and Gonzalez-Jimenez [30] proposed solving the 3D registration problem with global optimality guarantees, where Lagrangian duality theory is exploited for convex tight relaxation. Zhou et al. [31] presented a robust optimization method for a feature-based registration that used a scaled Geman–McClure estimator as a cost metric in the optimization procedure to cope with incorrect correspondences. Their method showed faster and more robust performance that ICP and Go-ICP. However, this algorithm generally tends to degrade in terms of accuracy when a set of correspondences have a high fraction that are incorrect. In addition, Poreba et al. [32] presented a RANSAC-based algorithm that used line-to-line correspondences to estimate the initial transformation, and Lu et al. [2] developed a coarse-to-fine feature-matching algorithm with multi-scale local features for recognizing a specific object in 3D point clouds. 

In this paper, a global optimization framework is proposed to solve the feature-based registration problem in the presence of mismatched correspondences. The presented method estimates optimal transformation parameters and simultaneously distinguishes inliers (correct correspondences) from outliers (mismatched ones). It should be noted that our method seeks to maximize the number of correct correspondences that satisfy the pose estimate, whereas traditional methods, such as ICP, seek to minimize the Root Mean Square Error. In addition, it should be noted that the lower bounding function in branch-and-bound techniques differs from one application to another since the general solution to the lower bounding function for a nonlinear problem does not exist. Our contribution is the mathematical formulation of 3D registration to manage outliers in a globally optimal manner and the formulation of a lower bounding function when applying a branch-and-bound algorithm specifically to a 3D Euclidean registration problem, with both rotation and translation, in the presence of mismatched correspondences. 

The remainder of this paper is organized as follows: Section 2 presents a MaxFS formulation of 3D registration, experimental results are provided in Section 3, and conclusions are presented in Section 4. 

## 2. Mixed-Integer Linear-Programming (MILP)-Based Branch-and-Bound Algorithm for 3D Euclidean Registration

**Figure 1:**
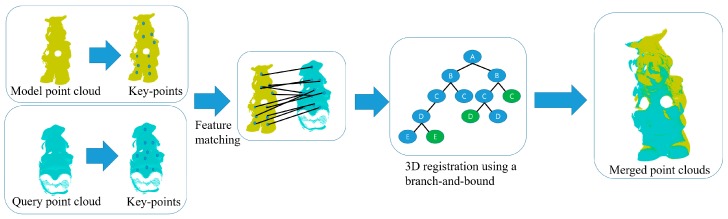
The pipeline of the 3D feature-based registration using the proposed method.

In this section, we propose a robust 3D Euclidean registration framework based on a branch-and-bound technique, and the pipeline of our 3D registration is shown in Figure 1. Our main contribution focuses the development of the branch-and-bound method for solving a coarse 3D registration problem, and the putative point-to-point correspondences established from the detected 3D features in the two range images are exploited as inputs in our proposed branch-and-bound algorithm. This section describes a mixed-integer linear-programming (MILP)-based branch-and-bound method, which is based on a Big-M formulation for solving the 3D Euclidean registration problem in the presence of mismatched correspondences.

### 2.1. 3D Euclidean Pairwise Registration with Mismatched Correspondences

Consider two point cloud sets. Correspondences between these sets are established by 3D local feature matches, and the corresponding matches are used as inputs to our algorithm. Given a set of point-to-point correspondences, $p_{i}={\left[\begin{array}{ccc} x_{i} & y_{i} & z_{i} \end{array}\right]}^{T}$ and $p_{i}^{\prime }={\left[\begin{array}{ccc} x_{i}^{\prime } & y_{i}^{\prime } & z_{i}^{\prime } \end{array}\right]}^{T},\;i=1,\ldots ,n\;\mathrm{in}\;{ℜ}^{3}$, the registration problem is finding a rotation parameter, ***R*** ∈ *SO*(3), and translation parameter, ***T*** ∈ **ℜ**^3^, that satisfies the rigid-body transformation:

(1) $$p_{i}^{\prime }=Rp_{i}+T,\;\forall \iota ,$$

where the 3 × 3 rotation matrix, ***R***, derived from the unit quaternion, $q={\left[\begin{array}{cccc} q_{0} & q_{1} & q_{2} & q_{3} \end{array}\right]}^{T}$, is represented as:

(2) $$R=\left[\begin{array}{ccc} q_{0}^{2}+q_{1}^{2}-q_{2}^{2}-q_{3}^{2} & 2\left(q_{1}q_{2}-q_{0}q_{3}\right) & 2\left(q_{1}q_{3}+q_{0}q_{2}\right)\\ 2\left(q_{1}q_{2}+q_{0}q_{3}\right) & q_{0}^{2}-q_{1}^{2}+q_{2}^{2}-q_{3}^{2} & 2\left(q_{2}q_{3}-q_{0}q_{1}\right)\\ 2\left(q_{1}q_{3}-q_{0}q_{2}\right) & 2\left(q_{2}q_{3}+q_{0}q_{1}\right) & q_{0}^{2}-q_{1}^{2}-q_{2}^{2}+q_{3}^{2} \end{array}\right]$$

Solving the 3D Euclidean registration between the two point clouds poses a challenge because of the non-convexity of the rotation parameter, ***R*** ∈ *SO*(3), and the outliers.

Outliers should be removed for the accurate estimation of parameters. Therefore, the present objective is to find the maximum number of feasible correspondences and the optimal pose estimation of ***R*** and ***T*** so they satisfy the inequality in Equation (3) for a predefined maximum tolerance, *ε*;

(3) $${\left|\;Rp_{i}+T-p_{i}^{\prime }\right|}_{\infty }\leq \varepsilon ,\;\forall i,$$

and this pose estimation problem to deal with outliers (spurious correspondences) could be formulated as a maximum feasible cardinality problem as follows:

(4) $$\begin{array}{l} \underset{R,\;T}{\max}\;\;\mathrm{card}\left(S_{I}\right)\\ s.t.\;\;{\left|Rp_{i}+T-p_{i}^{\prime }\right|}_{\infty }\leq \varepsilon ,\;\;\forall \;i\in S_{I}\subseteq S, \end{array}$$

where the cardinality of ***S_I_***, card (***S_I_***), corresponds to the number of feasible correspondences; and the set of point-to-point correspondences, ***S***, is split into two mutually exclusive subsets: the feasible subset, ***S_I_***, which consists of inliers (correct correspondences) and the infeasible subset, ***S_o_***, which consists of outliers (incorrect correspondences). The objective of the maximum feasible cardinality problem in Equation (4), is to find the largest cardinality set for which the constraints are feasible and to estimate the pose estimation of ***R*** and ***T***, simultaneously, while spurious correspondences have no effect. The problem of finding the maximum feasible cardinality subset, ***S_I_***, in an infeasible set, ***S***, is known as a MaxFS problem, and one of the most renowned solutions to which is the Big-M method. 

The Big-M formulation required to solve Equation (4) is constructed by adding the product of a positive value, *M*, and a binary variable, *s_i_*, to each of the inequalities. The 3D rigid-body registration problem to manage outliers is formulated as a mixed integer non-linear programming (MINLP) problem as follows:

(5) $$\begin{array}{r} \min\;\sum_{i=1}^{n}s_{i},\;s.t.\;{\left|Rp_{i}+T-p_{i}^{\prime }\right|}_{\infty }\leq \varepsilon +Ms_{i}\;,\;\forall \iota ,\\ \sum_{j=0}^{3}q_{j}^{2}=1,\;\sum_{j=1}^{3}q_{j}^{2}\leq 1\;,\;q_{0}\geq 0\;,\\ q\in {\left[-1,1\right]}^{4},\;T\in {ℜ}^{3},\;s\in {\left\{0,1\right\}}^{n}, \end{array}$$

where *n* indicates the number of correspondences; tolerance, *ε*, is the residual threshold; *s_i_* denotes the *i*th element of the slack variable, ***s***; and *M* represents a large positive number that converts infeasible inequalities into feasible ones when *s_i_* = 1. If the given inequality constraint is infeasible, the corresponding binary variable, *s_i_*, can be either one or zero. Nevertheless, minimizing the objective function in Equation (5) forces *s_i_* to one. Therefore, the case in which *s_i_* = 0 indicates that the corresponding inequalities are feasible. If *s_i_* = 1, the corresponding constraints are automatically deactivated; hence, the corresponding constraints have no effect on the registration result. The inequality constraint $q_{0}\geq 0$ is imposed to remove the sign ambiguity, and constraints $\sum_{j=0}^{3}q_{j}^{2}=1$ and $\sum_{j=1}^{3}q_{j}^{2}\leq 1$ represent a unit-norm quaternion constraint. In addition, the constraint equation (${\left|Rp_{i}+T-p_{i}^{\prime }\right|}_{\infty }\leq \varepsilon +Ms_{i}$) of Equation (5) can now be expressed by the following six linear inequalities:

(6) $$\begin{array}{l} -\varepsilon -Ms_{i}\leq r_{0}p_{i}+t_{0}-x_{i}^{\prime },\;r_{0}p_{i}+t_{0}-x_{i}^{\prime }\leq \varepsilon +Ms_{i},\\ -\varepsilon -Ms_{i}\leq r_{1}p_{i}+t_{1}-y_{i}^{\prime },\;r_{1}p_{i}+t_{1}-y_{i}^{\prime }\leq \varepsilon +Ms_{i},\\ -\varepsilon -Ms_{i}\leq r_{2}p_{i}+t_{2}-z_{i}^{\prime },\;r_{2}p_{i}+t_{2}-z_{i}^{\prime }\leq \varepsilon +Ms_{i}, \end{array}$$

where *t_j_* is the *j*th element of translation vector ***T***; ***r**_j_* is the *j*th row vector of the 3 × 3 rotation matrix, ***R***; and the binary variable, *s_i_*, connects the six linear constraints to the *i*th correspondence.

Nonetheless, to the best of our knowledge, there exists no general MINLP solver with guaranteed global optimality. We, therefore, propose a MILP-based branch-and-bound method to solve Equation (5) that yields the registration parameters estimated with the largest number of inliers in a globally optimal manner. 

### 2.2. MILP-Based Branch-and-Bound Method for 3D Registration 

We adopt a branch-and-bound strategy to solve the MINLP formulation of the 3D rigid-body registration problem, which maximizes the cardinality of the feasible set that satisfies the inequality and equality constraints in Equation (5). The branch-and-bound algorithm requires an upper bounding function, Φ*_ub_*(*D*), and a lower bounding function, Φ*_lb_*(*D*), with respect to the optimal value, Φ*_min_*(*D*), over any $D\subseteq D_{init}$. It should be noted that Φ*_ub_*(*D*) and Φ*_lb_*(*D*) are easier to compute than Φ*_min_*(*D*), and these bounding functions should satisfy the following condition: Φ*_lb_*(*D*) ≤ Φ*_min_*(*D*) ≤ Φ*_ub_*(*D*). Our branch-and-bound relies on two subroutines that, respectively, compute the lower and upper bounds of the optimal value over a given parameter space. The upper and lower bounds of the proposed branch-and-bound method will be obtained via local optimization and MILP relaxation, respectively. The MILP-based branch-and-bound method used to solve the 3D Euclidean registration problem with outliers is summarized in Algorithm 1.

**Algorithm 1** MILP-based branch-and-bound method for 3D registration with outliersInput: Initial rotation parameter bounding-box *D*_0_ for ***q***.Output: Optimal transformation parameter, R^*^ and T^*^, and the largest inlier set.1:    Initialize *k* = 0, **Ω**_0_ = {*D*_0_}, *L_0_* = 0, *U_0_* = ∞.2:    while *L_k_* > *U_k_*, then {3:            Choose *D* ∈ **Ω***_k_*, for which Φ*_lb_*(*D*) = *L_k_*.4:            Bisect *D* along one of its longest edges into {*D*_1_, *D*_2_}.5:            **Ω**_*k*+1_ ← {**Ω***_k_*\*D*}∪{*D*_1_, *D*_2_}.6:            *k* ← *k +* 1.7:            Compute the lower bounds, {Φ*_lb_*(*D*_1_), Φ*_lb_*(*D*_2_)}, and upper bounds, {Φ*_ub_*(*D*_1_), Φ*_ub_*(*D*_2_)}.8:            Update the global lower and upper bounds: *L_k_* and *U_k_*.9:            Prune **Ω***_k_* by discarding $\forall D\in \Omega_{k}$ if Φ*_lb_*(*D*) >*U_k_*}.

In Algorithm 1, *k* represents the iteration index, **Ω***_k_* denotes the list of sub-domains, and Φ*_ub_*(*D*) and Φ*_lb_*(*D*) are the upper and lower bounds over any *D*⊆*D*_0_, respectively. After the *k*th iteration of a branch-and-bound search tree, the associated lower and upper bounds of the globally optimal value, $f^{*}$, can be found as:

(7) $$L_{k}=\underset{D\in \Omega }{\min}\;\Phi_{lb}\left(D\right),\;U_{k}=\underset{D\in \Omega }{\min}\;\Phi_{ub}\left(D\right),$$

At the *k*th iteration step, the globally optimal value, $f^{*}$ and the global lower and upper bounds have the following relationship: $L_{k}\leq f^{*}\leq U_{k}$. Thus, our algorithm is terminated and the global solution is obtained when two global bounds converge. At each iteration, we exclude some subspace, *D* ∈ Ω*_k_* , in the branch-and-bound search tree that satisfies, Φ*_lb_*(*D*) > *U_k_* , since there is no possibility that $f^{*}$ is found from those subspaces.

A modified version of the branching strategy of a partial branch-and-bound is adopted [20]. Instead of branching in the entire parameter space (***R***, ***T***, and ***s***), only the parameter space of the quaternion (***R***) is divided; thus, the search space of our branch-and-bound algorithm is four-dimensional over the rotation parameter. In addition, a best-first search strategy for node selection in a branch-and-bound search tree is adopted, in this paper, due to memory space and computational efficiency, and this strategy selects one with the globally lowest bound value (i.e., the node that satisfies Φ*_lb_*(*D*) = *L_k_*, which has a relatively high possibility of containing the global minimum). Once the parameter space, *D*, to divide by a best-first search is chosen, it is divided into two sub-spaces, *D*_1_ and *D*_2_, along one of its longest intervals.

We formulate the lower bound registration problem as a MILP problem by relaxing the unit norm equality and quadratic inequality constraints on the quaternion. The lower bound, Φ*_lb_*(*D*), of the optimal value over a given domain, *D*, at each iteration can be obtained from the relaxed MILP in Equation (5), where the MILP guarantees the global optimality of its solution. First, we lead the relaxed MILP from MINLP by substituting a bilinear term, *xy*, where *x* ∈ [*x^lb^*, *x^ub^*] and *y* ∈ [*y^lb^*, *y^ub^*], with the single variable, *v*. Then, over- and under-envelope functions in Equation (8) for a bilinear term are constructed by applying McCormick’s relaxations, which is represented by the convex and concave functions having the tightest linear bound [33], as follows:

(8) $$\begin{array}{l} concave(xy)=\min\left(x^{lb}y+y^{ub}x-x^{lb}y^{ub},\;x^{ub}y+y^{lb}x-x^{ub}y^{lb}\right),\\ convex(xy)=\max\left(x^{lb}y+y^{lb}x-x^{lb}y^{lb},\;x^{ub}y+y^{ub}x-x^{ub}y^{ub}\right),\\ convex(xy)\leq v\leq concave(xy). \end{array}$$

This relaxation is represented by four linear inequalities:

(9) $$\begin{array}{l} v\geq x^{lb}y+y^{lb}x-x^{lb}y^{lb},\\ v\geq x^{ub}y+y^{ub}x-x^{ub}y^{ub},\\ v\leq x^{ub}y+y^{lb}x-x^{ub}y^{lb},\\ v\leq x^{lb}y+y^{ub}x-x^{lb}y^{ub}. \end{array}$$

Second, a square term, *x*^2^, where *x* ∈ [*x^lb^*, *x^ub^*] is replaced with the single variable, *u*, and over- and under-envelope functions of this square term are defined as:

(10) $$\begin{array}{l} concave(x^{2})=\left(x^{lb}+x^{ub}\right)x-x^{lb}x^{ub},\\ convex(x^{2})=\min\left(2x^{lb}x-{\left(x^{lb}\right)}^{2},\;2x^{ub}x-{\left(x^{ub}\right)}^{2}\right),\\ convex(x^{2})\leq u\leq concave(x^{2}), \end{array}$$

This linear relaxation is represented by three linear inequalities:

(11) $$\begin{array}{l} u\geq 2x^{lb}x-{\left(x^{lb}\right)}^{2},\\ u\geq 2x^{ub}x-{\left(x^{ub}\right)}^{2},\\ u\leq \left(x^{lb}+x^{ub}\right)x-x^{lb}x^{ub}. \end{array}$$

After the linear relaxation of the square and bilinear terms in Equation (5), the lower bound, Φ*_lb_*(*D*) is obtained by solving the following MILP problem:

(12) $$\begin{array}{l} \min\sum_{i=1}^{n}s_{i}\\ s.t.\;{\left|Rp_{i}+T-p_{i}^{\prime }\right|}_{\infty }\leq \varepsilon +Ms_{i},\;\forall \iota ,\\ \;R=\left[\begin{array}{ccc} u_{0}^{}+u_{1}^{}-u_{2}^{}-u_{3}^{} & 2\left(v_{3}-v_{2}\right) & 2\left(v_{4}+v_{1}\right)\\ 2\left(v_{3}+v_{2}\right) & u_{0}^{}-u_{1}^{}+u_{2}^{}-u_{3}^{} & 2\left(v_{5}-v_{0}\right)\\ 2\left(v_{4}-v_{1}\right) & 2\left(v_{5}+v_{0}\right) & u_{0}^{}-u_{1}^{}-u_{2}^{}+u_{3}^{} \end{array}\right],\\ \;u_{0}+u_{1}+u_{2}+u_{3}=1,\;u_{1}+u_{2}+u_{3}\leq 1,\;q_{0}\geq 0\;,\\ \;\mathrm{convex}\left(q_{0}^{2}\right)\leq u_{0}^{}\leq \mathrm{concave}\left(q_{0}^{2}\right),\;\mathrm{convex}\left(q_{1}^{2}\right)\leq u_{1}^{}\leq \mathrm{concave}\left(q_{1}^{2}\right),\;\mathrm{convex}\left(q_{2}^{2}\right)\leq u_{2}^{}\leq \mathrm{concave}\left(q_{2}^{2}\right),\\ \;\mathrm{convex}\left(q_{3}^{2}\right)\leq u_{3}^{}\leq \mathrm{concave}\left(q_{3}^{2}\right),\;\mathrm{convex}\left(q_{0}q_{1}\right)\leq v_{0}\leq \mathrm{concave}\left(q_{0}q_{1}\right),\;\mathrm{convex}\left(q_{0}q_{2}\right)\leq v_{1}\leq \mathrm{concave}\left(q_{0}q_{2}\right),\\ \;\mathrm{convex}\left(q_{0}q_{3}\right)\leq v_{2}\leq \mathrm{concave}\left(q_{0}q_{3}\right),\;\mathrm{convex}\left(q_{1}q_{2}\right)\leq v_{3}\leq \mathrm{concave}\left(q_{1}q_{2}\right),\;\\ \;\mathrm{convex}\left(q_{1}q_{3}\right)\leq v_{4}\leq \mathrm{concave}\left(q_{1}q_{3}\right),\;\mathrm{convex}\left(q_{2}q_{3}\right)\leq v_{5}\leq \mathrm{concave}\left(q_{2}q_{3}\right),\\ \;q\in \left[q^{lb},\;q^{ub}\right],\;u\in \left[u^{lb},\;u^{ub}\right],\;v\in \left[v^{lb},\;v^{ub}\right],\;T\in {ℜ}^{3},\;s\in {\left\{0,1\right\}}^{n}, \end{array}$$

where *u_i_* and *v_i_* are the *i*th element of the additive variables, ***u*** ∈ **ℜ**^4^ and ***v*** ∈ **ℜ**^6^, and are used to linearize the square and bilinear terms in Equation (5).

To compute the upper bound, Φ*_ub_*(*D*), the absolute orientation algorithm [15] is employed, where only the candidate inliers detected from the relaxed MILP are used as an input. The upper bound is simply calculated by counting the number of correspondences among those that do not satisfy the condition ${\left|R^{*}p_{i}+T^{*}-p_{i}^{\prime }\right|}_{\infty }\leq \varepsilon$, where $R^{*}$ and $T^{*}$ are rotation and translation estimates obtained from the absolute orientation algorithm.

## 3. Experiments and Discussion

**Figure 2:**
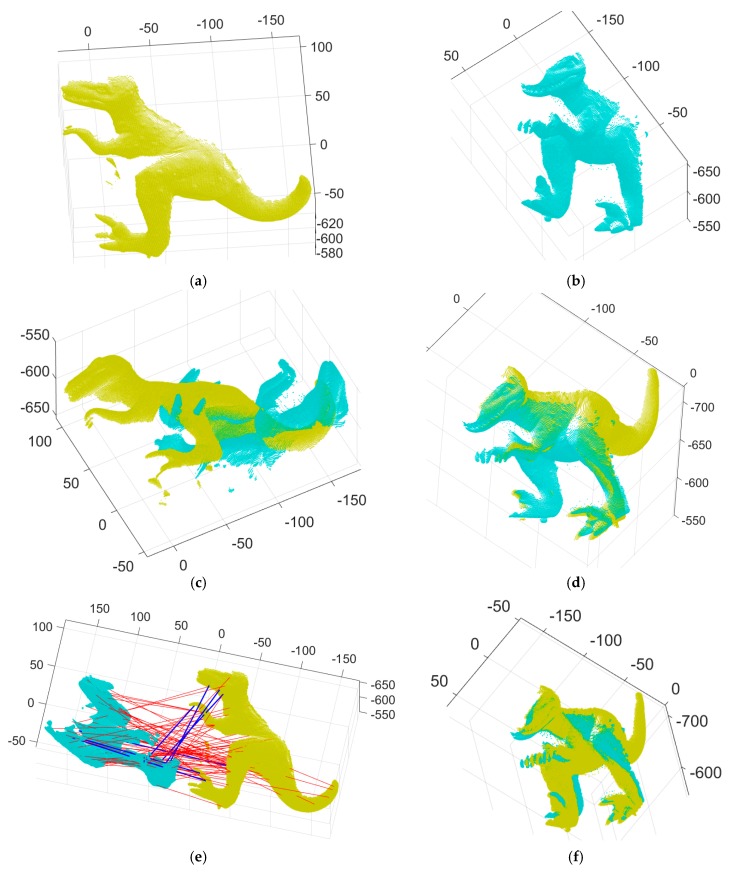

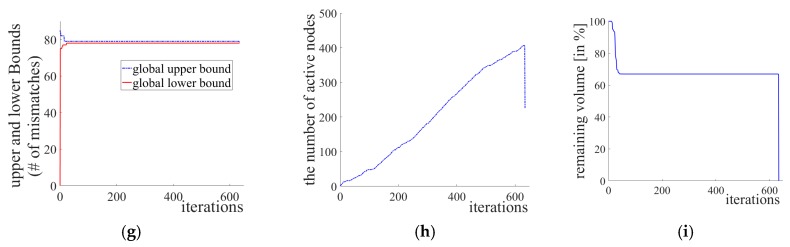
3D registration results for the proposed method: (**a**,**b**) two input range images, (**c**) initial pose of two input data (**a**,**b**,**d**) range images aligned by our branch-and-bound, (**e**) the correct and incorrect matches detected by the proposed method, where blue and red lines denote a correct one and a mismatch, respectively, (**f**) the refined registration result of (**d**) by iterative closest point (ICP), (**g**) convergence of the global lower and upper bounds of our branch-and-bound, (**h**) number of active nodes in our branch-and-bound, and (**i**) the remaining volume of our branch-and-bound search space.

**Figure 3:**
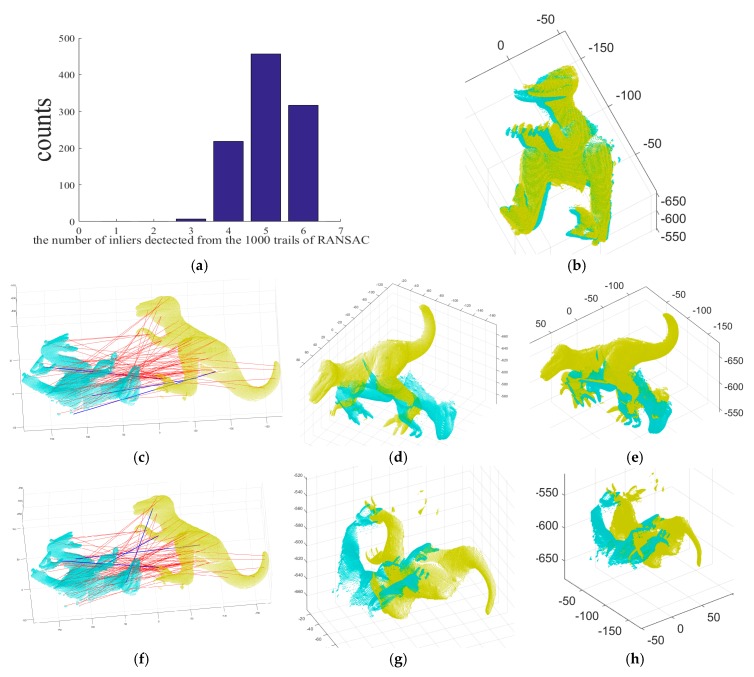

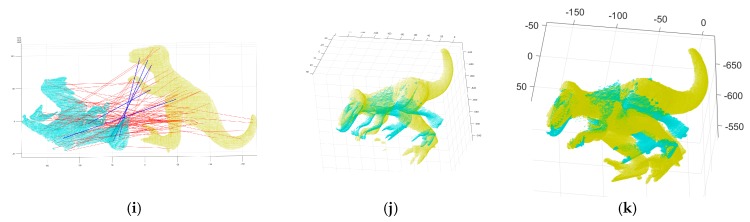
3D registration results for the random sample consensus (RANSAC)-based method and some failure cases: (**a**) histogram of the number of inliers classified by RANSAC for two input data of Figure 2a,b, (**b**) the refined registration obtained by applying the ICP to range images aligned with the 6 inliers detected by the RANSAC-based method, (**c**,**f**,**i**) the correct and incorrect matches detected by the RANSAC-based method, where blue and red lines denote a correct one and a mismatch, respectively, (**d**,**g**,**j**) range images aligned with the 3, 4, and 5 inliers detected in (**c**,**f**,**i**), respectively, (**e**,**h**,**k**) the refined registration result of (**d**,**g**,**j**) by ICP, respectively.

**Figure 4:**
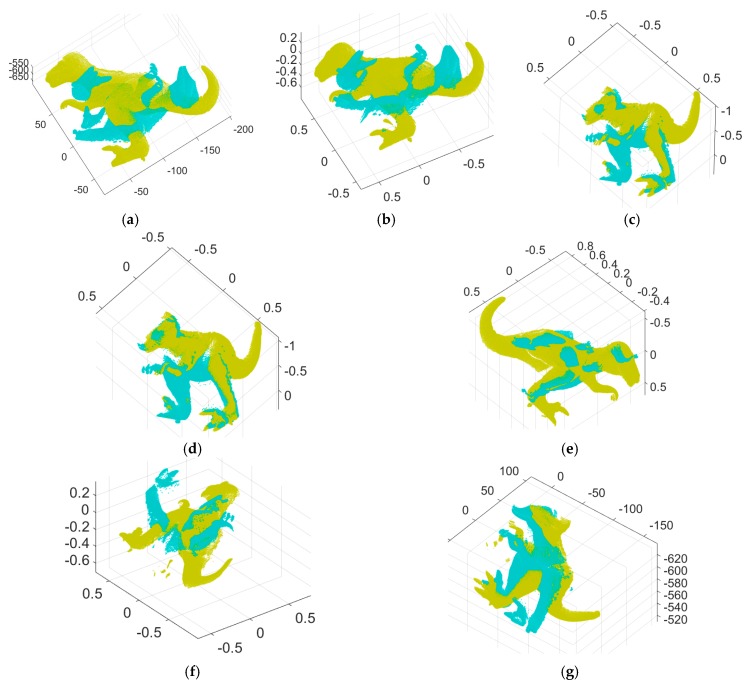
3D registration results for the dense registration algorithms: (**a**) range images aligned by ICP [7] for Figure 2a,b, (**b**) range images aligned by globally optimal (Go)-ICP [24], (**c**–**f**) range images aligned by trimmed Go-ICP, with respective trimming percentages of 20%, 30%, 50%, and 80%, (**g**) registration result by fast global registration [31].

**Figure 5:**
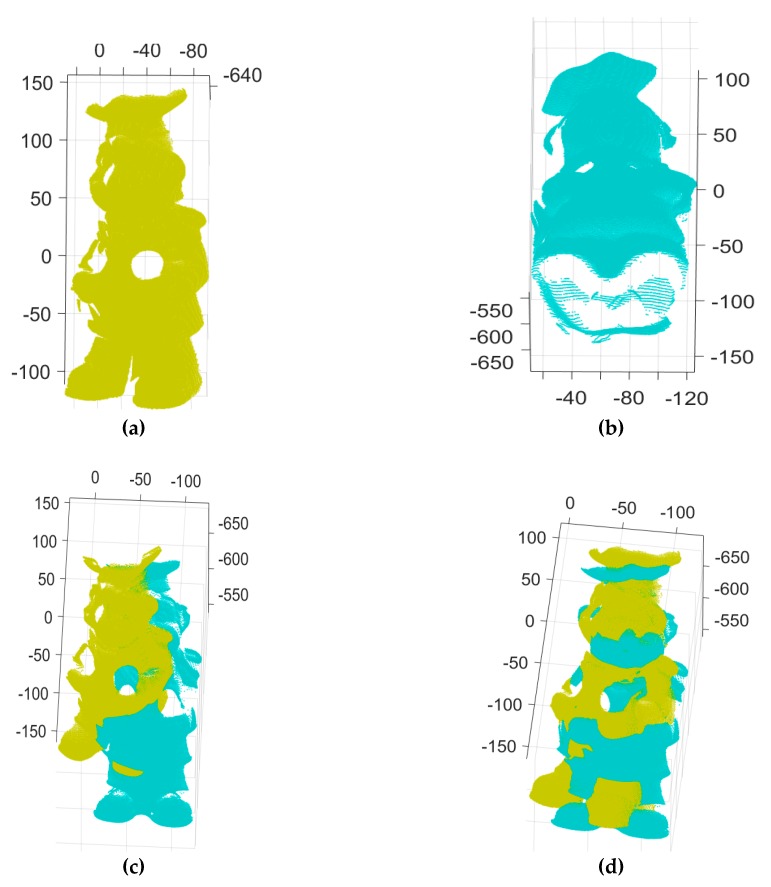

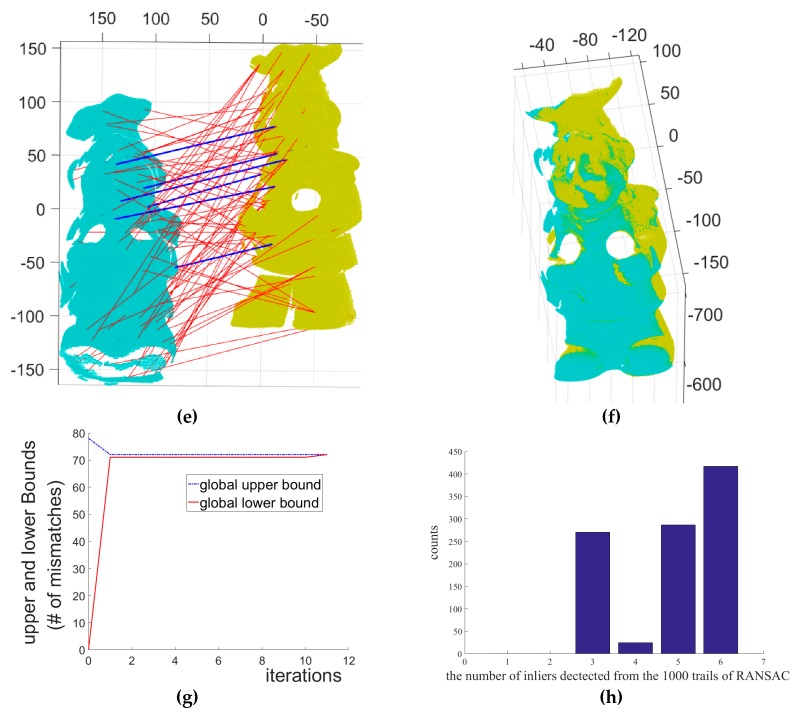
3D registration results for the proposed method and others: (**a**,**b**) two input range images, (**c**) initial pose of two input data (**a**,**b**,**d**) range images aligned by ICP, (**e**) the correct and incorrect matches detected by the proposed method, where blue and red lines denote a correct one and a mismatch, respectively, (**f**) range images aligned by our branch-and-bound, (**g**) convergence of the global lower and upper bounds of our branch-and-bound, and (**h**) histogram of the number of inliers classified by RANSAC.

**Figure 6:**
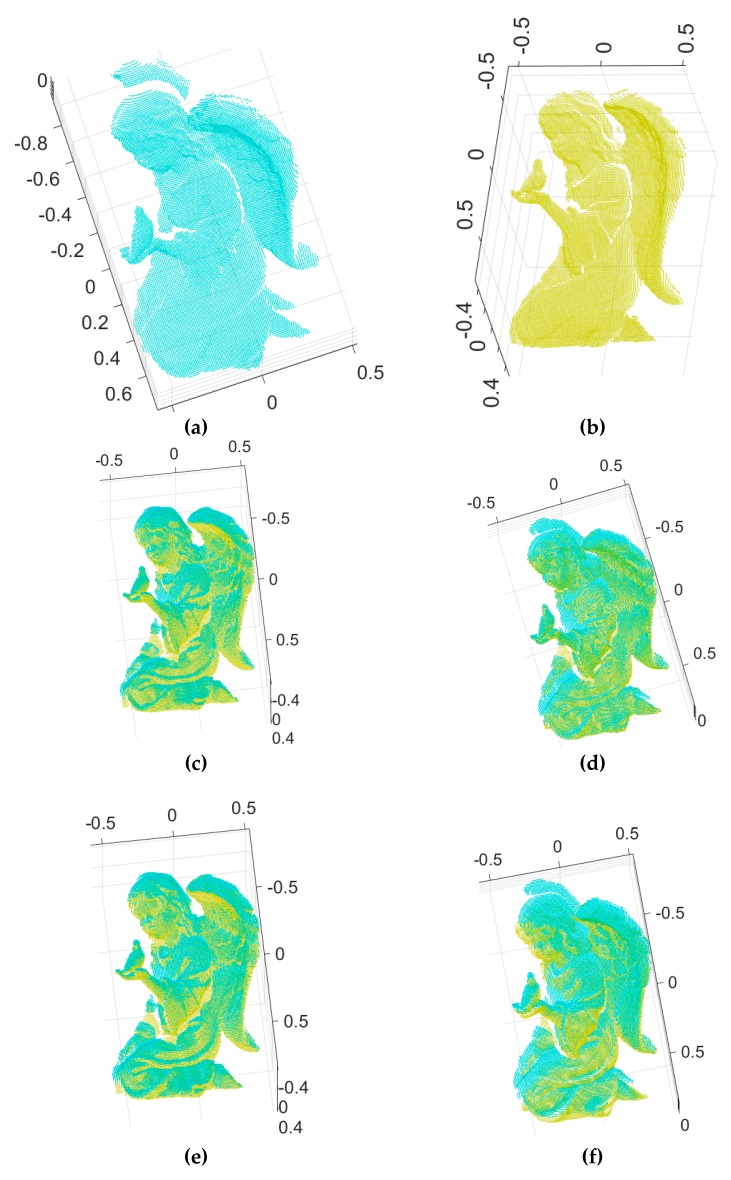

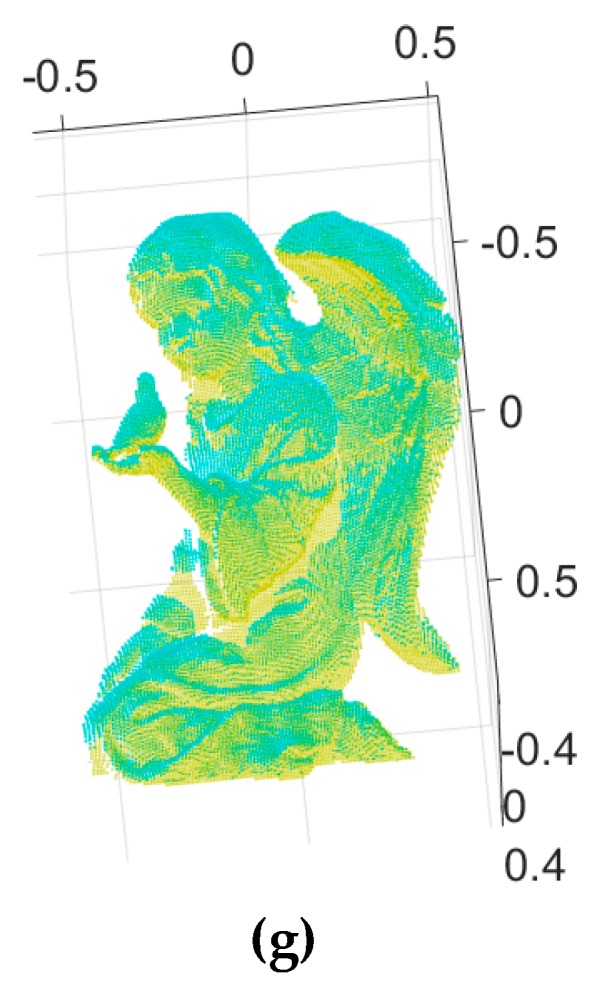
3D registration results for the proposed method and others: (**a**,**b**) two input synthetic range images with no noise, (**c**–**e**) range images aligned by Go-ICP, trimmed Go-ICP, and fast global registration, respectively, (**f**) range images aligned by our branch-and-bound, (**g**) the refined registration result of (**f**) by ICP.

**Figure 7:**
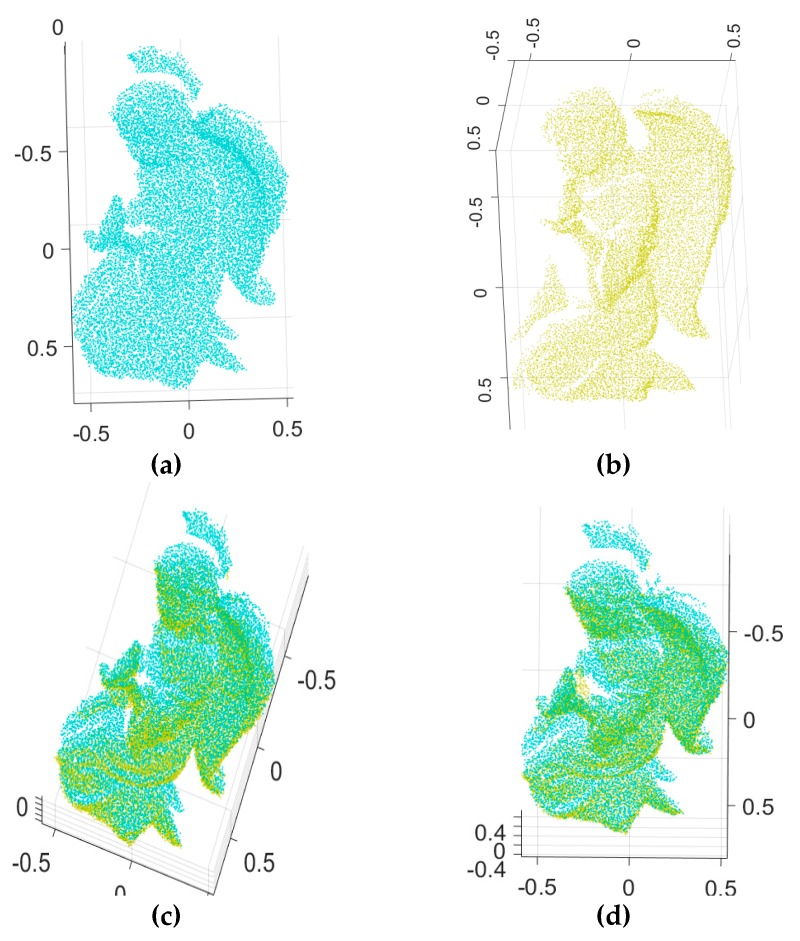

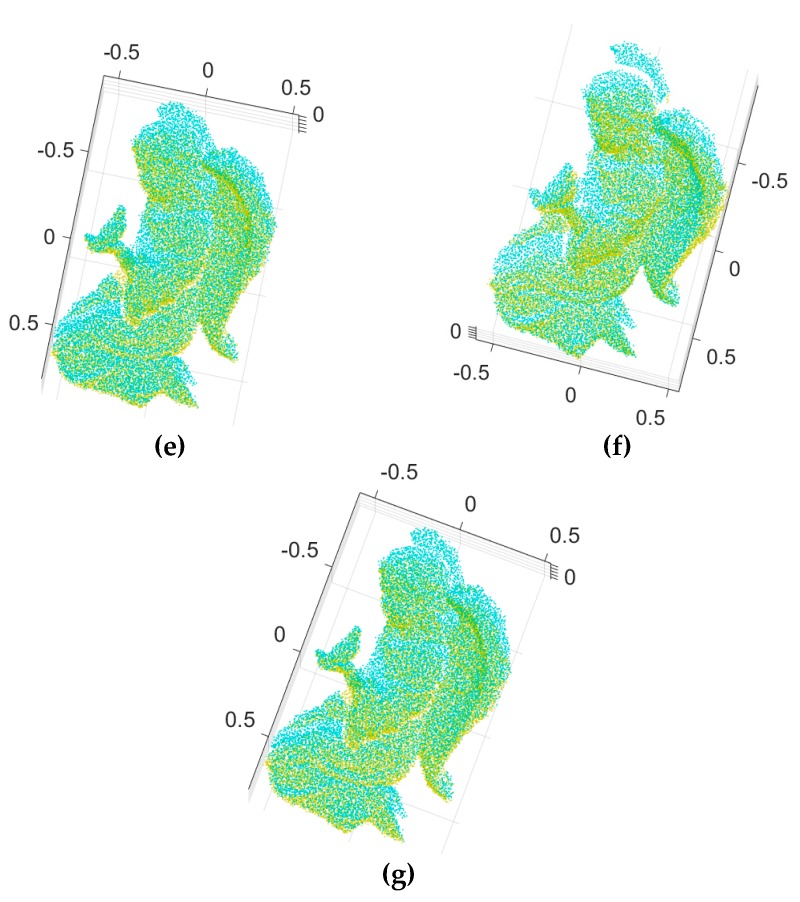
3D registration results for the proposed method and others: (**a**,**b**) two input synthetic range images with added Gaussian noise (*σ* = 0.0025), (**c**–**e**) range images aligned by Go-ICP, trimmed Go-ICP, and fast global registration, respectively, (**f**) range images aligned by our branch-and-bound, (**g**) the refined registration result of (**f**) by ICP.

We conducted 3D registration experiments with two pairs of range images to verify the accuracy of our algorithm and compare it to other methods. Our method was implemented using C++ with the Gurobi optimization package [34] to solve MILP, and all our experiments were performed on a desktop computer with an Intel i7-5820K 3.30 GHz processor and 64 GB of RAM. Figure 2, Figure 3, Figure 4, Figure 5, Figure 6 and Figure 7 show the two pairs of input range images and the 3D Euclidean registration results obtained using the five different methods. We employed three dense-data registration methods—ICP, Go-ICP, and the fast global registration algorithm proposed by Zhou et al. [31]—and two feature-based methods, one with RANSAC and the other with our global optimization algorithm. For the two feature-based methods (RANSAC-based and our MaxFS-based branch-and-bound algorithms), we used an intrinsic shape signature (ISS) key-point detector associated with a SHOT descriptor provided in the Point Cloud Library [14,35,36]. We set the tolerance to five times the average depth resolution of the point clouds. 

Figure 2, Figure 3 and Figure 4 show the two input range images of a T-Rex object and the 3D Euclidean registration results obtained using the five different methods. Figure 2a,b depict the two 2.5D range data of a T-Rex object from the University of Western Australia (UWA) range image database [37,38]. They were densely scanned with the Minolta Vivid 910 scanner to get 2.5D range images of two different directions. From the detected 3D features in the two range images, 85 putative point-to-point correspondences were established, as shown in Figure 2e. Figure 2c shows the initial pose of the input data of Figure 2a,b. Figure 2d,e show range images aligned, and the correct and incorrect matches classified by our MaxFS-based branch-and-bound, where the blue and red lines denote an inlier and an outlier, respectively. ICP has often been used to refine the registration results obtained by performing the coarse registration. Figure 2f shows the refined registration obtained by applying the ICP to Figure 2d*.* Figure 2g shows the convergence of the global lower and upper bounds of our branch-and-bound. As shown in Figure 2g, the gap between the two bounds is relatively large in the initial search stage of our branch-and-bound. Nevertheless, the gap decrease as the iteration count increases. The global lower and upper bounds converge after 634 iterations; moreover, 78 outliers and seven inliers were found from the 85 putative correspondences. Figure 2h presents the number of active nodes in the branch-and-bound search tree at each iteration step, indicating the proposed algorithm’s memory usage. Figure 2i shows the remaining volume of the quaternion parameter space as the proposed algorithm converges. 

Figure 3 shows the registration results achieved by the absolute orientation algorithm [15], with RANSAC, on the inputs of Figure 2a,b. This RANSAC-based method was tested with 1000 RANSAC trials and the maximum number of iterations was set to 10,000 for all trials. Figure 3a shows the histogram of obtained inliers, ranging from three to six. Although RANSAC is a well-established means of finding inliers, its results are not consistent, and it did not find all of the inliers. Figure 3b shows the refined registration obtained by applying the ICP to range images aligned with the six inliers detected by the RANSAC-based method. Figure 3c–k show some failure cases that are aligned with the 3, 4, and 5 inliers detected by the RANSAC. Figure 3c,f,i show the correct and incorrect matches detected by the RANSAC-based method, shown by blue and red lines, respectively. Figure 3d,g,j show the point clouds aligned with the 3, 4, and 5 inliers detected in Figure 3c,f,i, respectively. Figure 3e,h,k show the refined registration obtained by applying the ICP to Figure 3d,g,j, respectively.

Figure 4 shows the registration results achieved by the dense registration algorithms—ICP, Go-ICP, and the fast global registration algorithm. Figure 4a shows the range images aligned by the ICP algorithm, starting from the initial pose of the input data of Figure 2a,b, which fails to obtain the correct result. Figure 4b–f show range images aligned by Go-ICP and its trimmed version, some of which fail to obtain good results. Different results were obtained for different trimming percentages. It should be noted that the Go-ICP algorithm requires prior knowledge of the approximate ratio of outliers to obtain a reasonable alignment, as shown in Figure 4b–f, when given small overlapped point clouds. For Go-ICP, we used a preprocessing procedure to rescale the point clouds to be located within [−1, 1]^3^ to use the publicly available Go-ICP code provided by the original authors. Figure 4g shows range images aligned by the fast global registration algorithm, which operates on fixed dense correspondences. While this dense registration method requires no initial guess of the transformation, it does not always provide stable registration results. 

Figure 5 shows the two input range images of a chef object and the 3D Euclidean registration results obtained using the three different methods. Figure 5a,b depict the two 2.5D range data of a chef object from the UWA range image database. Figure 5c shows the initial pose of the input data of Figure 5a,b. Figure 5d shows the range images aligned by the ICP algorithm. From the detected 3D features in the two range images, 78 putative point-to-point correspondences were established, as shown in Figure 5e. Figure 5e shows the correct and incorrect matches classified by our MaxFS-based branch-and-bound, shown by blue and red lines, respectively, Figure 5f shows range images aligned by our MaxFS-based branch-and-bound, and Figure 5g shows the convergence of the global lower and upper bounds of our MaxFS-based branch-and-bound. The global lower and upper bounds converge after 11 iterations, as shown in Figure 5g, and 72 outliers and six inliers were found from the 78 putative correspondences. Figure 5h shows the histogram of obtained inliers, ranging from three to six, where the RANSAC-based method was tested with 1000 RANSAC trials and the maximum number of iterations was set to 10,000 for all trials. We performed the experiments on the Go-ICP, trimmed Go-ICP, and fast global registration using the publicly available codes provided by the original authors. The running times for ICP, fast global registration [31], and RANSAC were 3.263 s, 6.109 s, and 54.087 s for a pair of the T-Rex range images, respectively. It required more than a day to obtain each result of Go-ICP and trimmed Go-ICP, as shown in Figure 4b,f. The computation time of our method was 2.102 h and 9 min 16 s for the pairs of the T-Rex and chef range images, respectively.

To validate the robustness of our algorithm with respect to the Gaussian noise, we carried out experiments using synthetic range data that included the ground truth value and synthetic range data obtained from [39]. Figure 6 shows the two input range images of an angel object and the 3D Euclidean registration results obtained using the proposed method and others. Figure 6a,b depict the two 2.5D synthetic range data. Figure 6c–e show range images aligned by Go-ICP, trimmed Go-ICP, and fast global registration, respectively. Figure 6f shows range images aligned by our branch-and-bound and Figure 6g shows the refined registration obtained by applying the ICP to Figure 6f*.* Moreover, Figure 7 shows the two input range data with added Gaussian noise (*σ* = 0.0025, where *σ* is the standard deviation of the Gaussian distribution) of an angel object and the 3D Euclidean registration results obtained using the proposed method and others.

Table 1 shows the average Root Mean Square (RMS) error achieved by our method and dense registration algorithms on synthetic range images of an angel object. In general, the dense registration algorithm, with a good initial guess, produces a more accurate registration result with a lower error level than feature-based coarse registration. Therefore, ICP is employed to refine the registration quality as a post-procedure, which is common in the registration process.

Table 2 shows the run times of our method for the number of tentative correspondences of three range image pairs ranging from 10 to 80, with intervals of 10. The computation time of our method increased exponentially as more data points were considered; therefore, it was dependent on the size of the data and outlier ratios. Our method was, thus, relatively slow because it relied on integer programming with a high computational cost. Nevertheless, our global optimization algorithm produced accurate and consistent registration results against severe outliers. 

## 4. Conclusions

In this paper, we presented the MaxFS-based 3D range data registration algorithm, which guarantees globally optimum results in the presence of severe outliers. The pairwise registration was formulated as a nonlinear integer problem and was solved by an MILP-based branch-and-bound. Our method required no initial alignment and produced reliable registration results, even when the geometric overlap between the data sets was very small, or when only a few correct correspondences were given between the detected features. The smaller the gap was between the lower bound function and the original problem, the faster the branch-and-bound algorithm converged. Hence, future research should include the development of a tighter lower bound function for the objective function of the registration problem.

## Figures and Tables

**Table 1:** Average Root Mean Square (RMS) error achieved by our method and dense registration algorithms on synthetic range images.

	*σ* = 0	*σ* = 0.0025
Go-ICP	3.1 × 10^−5^	2.15 × 10^−4^
Trimmed Go-ICP	1.9 × 10^−5^	1.37 × 10^−4^
Fast global registration	1.13 × 10^−4^	2.66 × 10^−4^
Our method	1.3919 × 10^−2^	7.962 × 10^−3^
Our method refined by ICP	1.85 × 10^−4^	1.64 × 10^−4^

**Table 2:** Run times of our method with respect to the different numbers of putative correspondences for the different range image pairs (unit: seconds).

**Number of putative correspondences**	10	20	30	40	50	60	70	80
**Average run-time**	7.41	66.09	107.30	287.47	434.74	1103.9	2502.3	3449.4
